# Why Does Positive Mental Health Buffer Against Psychopathology? An Exploratory Study on Self-Compassion as a Resilience Mechanism and Adaptive Emotion Regulation Strategy

**DOI:** 10.1007/s10608-016-9774-0

**Published:** 2016-04-09

**Authors:** Hester R. Trompetter, Elian de Kleine, Ernst T. Bohlmeijer

**Affiliations:** Centre for eHealth and Wellbeing Research, Department of Psychology, Health and Technology, University of Twente, P.O. Box 217, 7500 AE Enschede, The Netherlands

**Keywords:** Self-compassion, Positive mental health, Psychological wellbeing, Resilience, Psychopathology

## Abstract

Growing evidence suggests that positive mental health or wellbeing protects against psychopathology. How and why those who flourish derive these resilient outcomes is, however, unknown. This exploratory study investigated if *self*-*compassion*, as it continuously provides a friendly, accepting and situational context for negative experiences, functions as a resilience mechanism and adaptive emotion regulation strategy that protects against psychopathology for those with high levels of positive mental health. Participants from the general population (n = 349) provided measures at one time-point on positive mental health (MHC-SF), self-compassion (SCS-SF), psychopathology (HADS) and negative affect (mDES). Self-compassion significantly mediated the negative relationship between positive mental health and psychopathology. Furthermore, higher levels of self-compassion attenuated the relationship between state negative affect and psychopathology. Findings suggest that especially individuals with high levels of positive mental health possess self-compassion skills that promote resilience against psychopathology. These might function as an adaptive emotion regulation strategy and protect against the activation of schema related to psychopathology following state negative affective experiences. Enhancing self-compassion is a promising positive intervention for clinical practice. It will not only impact psychopathology through reducing factors like rumination and self-criticism, but also improve positive mental health by enhancing factors such as kindness and positive emotions. This may reduce the future risk of psychopathology.

## Introduction

There is growing interest in the field of psychology in positive mental health defined as the presence of optimal wellbeing in addition to an absence of psychopathology (Keyes 2005). Most used accounts of this view of mental health honor both hedonic and eudemonic traditions of wellbeing (Deci and Ryan 2008; Diener 1984; Ryff 1989). It includes both *feeling well* (e.g. happiness and positive affect) as well as *functioning well* in life psychologically (e.g. self-acceptance, environmental mastery, positive social relationships, and purpose in life) and socially (e.g. social acceptance and social integration) (Keyes 2002). There is accumulating evidence that psychopathology and positive mental health function along two different continua that are only moderately interrelated (Huppert and Whittington 2003; Keyes 2005; Lamers et al. 2011; Weich et al. 2011; Westerhof and Keyes 2009). The existence of this *dual*-*factor model of mental health* has several implications. Primarily, psychopathology and positive mental health do not function as exact opposites and must be seen as separate indicators of positive mental health. This makes positive mental health in itself a significant end-point of scientific study and intervention. Evidence that positive mental health over time functions as a *resilience resource* and protects against both physical and mental illness and disease even further amplifies its significance. For example, high levels of positive mental health are associated with heightened recovery and survival despite physical illness, and decreased cortisol levels and cardiovascular disease risk (Boehm and Kubzansky 2012; Lamers et al. 2012; Ryff 2013; Steptoe et al. 2009). In addition, positive mental health longitudinally protects against psychopathology at later moments in time (Grant et al. 2013; Keyes et al. 2010; Lamers et al. 2015; Wood and Joseph 2010). In sum, growing evidence exists on positive mental health as a resilience resource and contributor to adaptive functioning. This makes positive mental health highly relevant for clinical practice. In order to improve our knowledge and clinical interventions to enhance positive mental health and reduce psychopathology, we need to know more of the *working mechanisms* by which positive mental health enacts its potential as a resilience resource. One possible resilience mechanism in the relationship between positive mental health and psychopathology is self-compassion. Self-compassion is a relatively new concept in Western psychology, that is the self-directed equivalent to other-oriented compassion. It beholds a warm-hearted, caring, empathic and nonjudgmental orientation towards the self during times of suffering and failure, accompanied by a gentle motivation to alleviate this suffering (Gilbert 2009a; Neff 2003a). The most applied conceptualization of self-compassion (Neff 2003a, 2003b) includes three facets, being (1) self-kindness, the ability to be friendly and understanding towards the self during stress and failure as opposed to being self-criticizing, (2) common humanity, as the ability to recognize one’s suffering as part of the common, shared human experience in which failure and imperfections are normal and regular occurrences, as opposed to seeing suffering as personal and isolated and (3) mindfulness, the ability to take an open, accepting and nonjudgmental stance towards the self and suffering, as opposed to over-identification and fusion with the self. Recent studies suggest that people with higher levels of positive mental health have higher levels of self-compassion, and that a higher level of self-compassion may reduce the risk of psychopathology. For one, self-compassion can be seen as a positive human strength related to positive mental health. As it invokes kindness, a balanced and broadened awareness, and feelings of inter-connectedness and support, self-compassion contributes to the development of positive mental health resources such as positive emotions, self-acceptance, environmental mastery and positive social relations with others (Keyes 2005; Neff et al. 2007). A relatively large evidence-base supports these ideas. Studies showed that self-compassion is positively associated with factors related to positive mental health such as positive affect, life satisfaction, optimism, happiness, wisdom and personal initiative (Barnard and Curry 2011; Neff et al. 2007; Zessin et al. 2015). Other studies confirm that self-compassion relates negatively to negative affect, and psychopathology in the form of depression, anxiety and stress (Barnard and Curry 2011; Ehret et al. 2014; Hofmann et al. 2012; MacBeth and Gumley 2012; Muris et al. 2015). First interventional studies examining the feasibility and effectiveness of compassion-focused therapies—such as Compassion Focused Therapy (CFT) and Compassionate Mind Training (CMT) (Gilbert and Irons 2005; Gilbert 2009b)—revealed reductions in depression and anxiety in (non)clinical populations (Braehler et al. 2013; Gilbert and Procter 2006; Kelly et al. 2009). Findings in general confirm the relation of self-compassion with positive mental health on the one hand and with psychopathology on the other. In light of this study, that focuses on self-compassion as a resilience mechanism against psychopathology for those with high levels of positive mental health, we hypothesize that self-compassion mediates the previously described negative relationship between positive mental health and psychopathology (e.g. Lamers et al. 2015; Wood and Joseph 2010). This means that we expect individuals with high levels of positive mental health to possess more self-compassion skills, which they utilize in daily life during momentary stressful circumstances to buffer against the long-term development of psychopathology. However, an interesting and related question to the above hypothesis is *why* and via what processes self-compassion might bring this buffering effect about? Scholars have recently suggested that emotion regulation is one such mechanism. Self-compassionate individuals do not try to alter or escape from negative stressful experiences, but rather seem to modify the *context* in which these negative experiences occur. Within this context, self-compassionate individuals courageously expose themselves directly to the stressor at hand with feelings of care, support, openness, tolerance and equanimity. Existent literature suggests that self-compassion hereby serves as an antecedent-focused, adaptive emotion regulation strategy that mainly helps *positive cognitive reappraisal* and *acceptance* of negative situations (Allen and Leary 2010; Diedrich et al. 2014; Leary et al. 2007). Additionally, self-compassionate individuals seem to use less of maladaptive emotion regulation strategies related to depression and psychopathology such as *experiential avoidance* and escape of unwanted experiences, *thought suppression*, and *rumination* (Barnard and Curry 2011; Neff et al. 2007; Raes 2010). This means that, in relation to the development of depressive symptomatology and psychopathology, self-compassionate individuals might appraise negative situations and experiences as more momentary and controllable and as less aversive, thereby protecting against the in-depth activation or generation of depressogenic schema (Diedrich et al. 2014). However, at present not much literature exists on the functioning of self-compassion as emotion regulation strategy, and no research exists that assesses self-compassion as a resilience mechanism in relation to both positive mental health and psychopathology. The overall objective of this exploratory study is therefore to examine if self-compassion serves as a mechanism of resilience, and adaptive emotion regulation strategy, for people experiencing high levels of positive mental health. Using a cross-sectional dataset consisting of a convenience sample from the general Dutch population, we will first assess if self-compassion mediates the relationship between positive mental health and psychopathology. In a second set of analyses, we will then examine why these resilient outcomes are brought about, and study if self-compassion functions as an adaptive emotion regulation strategy for negative affective experiences as one potential pathway to resilience. Specifically, we hypothesize that self-compassion moderates the relationship between state negative affective experiences and psychopathology. We propose this hypothesis, as existing literature suggests that self-compassionate individuals might appraise negative experiences as more momentary, controllable and less aversive than less self-compassionate individuals, and this potentially provides a buffer against the in-depth activation or generation of schema related to psychopathology (Diedrich et al. 2014).

## Method

### Participants and Procedure

The study sample consisted of 349 participants who filled out an online survey conducted between November 2013 and May 2014. Ethical approval for this study was obtained from the Faculty of Behavioral Sciences Ethics Committee at the University of Twente in the Netherlands. Psychology graduate students, earning credits for a course in research methods, recruited participants. All students were instructed to recruit a convenience sample from their personal environment and to select a heterogeneous sample as possible. Individuals interested in participation were sent a unique link by e-mail to the online survey that was programmed in the online survey tool ‘Qualtrics’ (Provo, UT). Of the 349 participants, 64.5 % was female. Mean age of the participants was 32.88 (SD: 12.99), and ranged from 16 to 67 years of age. These and other descriptive characteristics can be found in Table 1.

**Table 1:** Descriptive characteristics and mean scores on questionnaires for all study participants (n = 349)

	%/M (SD)
Age [mean years (SD)]	32.88 (12.99)
Gender	
Female	64.5
Education	
Low	12.0
Intermediate	39.3
High	48.7
Marital status	
Not married	69.6
Married	27.8
Other (divorced, widowed)	2.6
Cultural background	
Dutch	72.5
Turkish	13.2
Mixed-dutch	8.0
Other	6.3
Religious background	
None	49.9
Roman catholic	14.3
Islam	14.9
Dutch reformed	6.6
Calvinist	4.6
Other	9.7
Daily activities	
Paid work	47.9
Student	37.8
Other	14.3
Health status	
Very good	25.5
Other	74.5
SCS-SF	40.89 (10.62)
HADS	8.55 (5.34)
MHC	57.71 (11.40)
mDES negative affect	16.27 (8.04)

### Measurement Instruments

The questionnaire included the following instruments:

#### Positive Mental Health

The *Mental Health Continuum*—*Short Form (MHC*-*SF)* is a 14-item self-report questionnaire developed to measure three dimensions of positive mental health (Keyes 2002; Lamers et al. 2011): emotional wellbeing, defined in terms of positive feelings (e.g. happiness and positive affect) and satisfaction with life (three items); psychological wellbeing; defined in terms of positive functioning in individual life (e.g. feelings of self-acceptance, environmental mastery, purpose in life, positive social relationships) (six items); and social wellbeing, defined in terms of positive functioning in community life (e.g. feelings of social contribution, social acceptance, and social integration) (five items). Participants rate the frequency of feelings in the past month on a scale from 1 (‘never’) to 6 (‘every day’). In this study the total MHC-SF was used, with higher scores indicating better positive mental health (range 14–84). Internal consistency in the present study was α = .90.

#### Self-Compassion

The *Self*-*Compassion Scale*—*Short Form (SCS*-*SF)* is a 12-item self-report questionnaire to assess self-compassion (Neff 2003b; Raes et al. 2011). This 12-item version is based on a validated Dutch 24-item version of the SCS (Neff and Vonk 2009) and has good psychometric properties. The SCS-SF includes questions on all components of self-compassion as identified by Neff: Self-kindness, self-judgement, common humanity, isolation, mindfulness, and over-identification. Items are each rated on a seven-point response scale from 1 (‘almost never’) to 7 (‘almost always’). Negatively worded items are mirrored to be able to compute a total self-compassion score, where higher scores indicate more self-compassion (range 12–84). Internal consistency in the present study was α = .81.

#### Psychopathology

The *Hospital Anxiety Depression Scale (HADS)* is a 14-item screening instrument for the presence of psychopathology in the form of anxiety (7 items) and depressive states (7 items) (Zigmond and Snaith 1983). Participants rate the frequency of feelings over the last week on a scale from 0 (‘not at all’) to 3 (‘very often’). As there is evidence for a strong single dimension of general psychopathology in the HADS (Spinhoven et al. 1997), in this study the total HADS was used with higher scores indicate higher indications for psychopathology (range 0–42). Internal consistency in the present study was α = .83.

#### Negative Affect

The *modified Differential Emotions Scale (mDES)* is a 16-item measure of state positive (eight items) and negative affective states (eight items) using mood adjectives (Izard 1977; Schaefer et al. 2010). Participants rated the intensity of feelings at the present moment on a scale from 1 (‘not at all’) to 7 (‘very intense’). Exploratory factor analysis (Maximum Likelihood, Varimax rotation) showed a clear two-factor structure consisting of a subscale on state negative and positive affect. For this study only the negative affect items were used. All eight negative mood adjectives (e.g. ‘afraid’, ‘anxious’, ‘angry’, ‘sad’, ‘guilty’) loaded ≥.59 on the first factor measuring state negative affect, and were summed into a total scale. Internal consistency of the scale in the present study was α = .89.

### Analysis

**Fig. 1:**
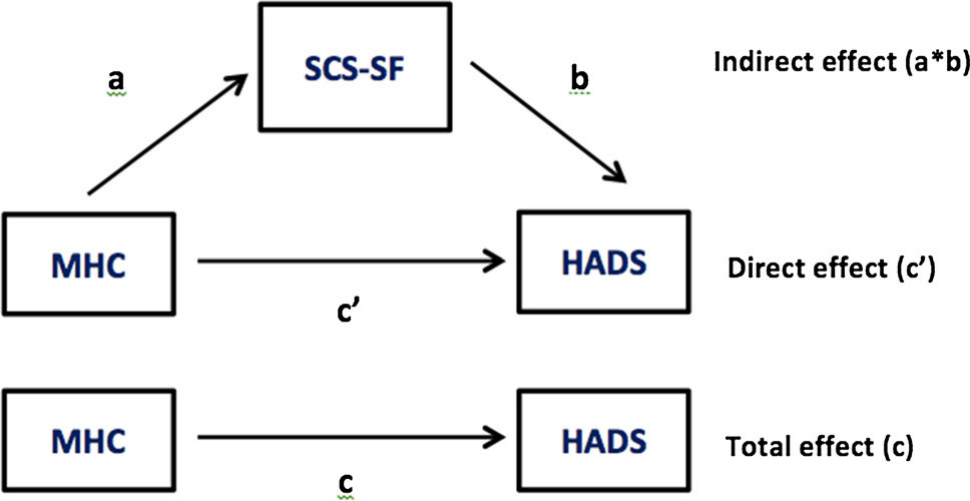
Mediation model. Total effect (c) = direct effect (c′) + indirect effect (a*b). The indirect effect (a*b) must be interpreted to assess if self-compassion (SCS-SF) significantly mediates the relationship between positive mental health (MHC) and psychopathology (HADS)

In total, 423 users were logged in Qualtrics. As 74 users did not start the questionnaire (but only, e.g., IP-addresses were logged), these users were removed from further analyses and not treated as participants. This resulted in an actual dataset of 349 participants. All statistical analyses were performed using SPSS 22.0 (IBM SPSS Statistics). First, missing values and outlier analyses were performed. No data was missing for any of the descriptive variables, except for health status and age. For variables containing missing values, on average 4.48 % of data was missing due to inconsistent reporting by participants. In total, 8 % of participants (n = 28) had one or more missing data points. Chi-square and independent *t* tests revealed that people with a lower age (F = 41.927, *p* = .000), lower educational level (χ^2^ = 4.942, *p* = .026) and who were not married (χ^2^ = 8.900, *p* = .003) were significantly more likely to have missing data. Further statistical analyses were controlled for these descriptive variables. Missing data were imputed using the Expectation–Maximization (EM) algorithm (Dempster et al. 1977). Outlier analyses revealed five potential outliers in either HADS (n = 2) or mDES (n = 3). As removal of these outliers did not alter the results of any analyses performed, only results with outliers included will be shown and discussed in the remainder of this article. Hereafter, correlation coefficients were calculated between all questionnaire variables (MHC-SF, SCS-SF, mDES negative affect, HADS) and descriptive variables in the study. First, following an exploratory inspection of frequency distributions, as meaningful and equally distributed two-category dummy variables were created for each descriptive variable: gender [female (1) vs. male], educational level [highly educated (1) vs. other], marital status [married (1) vs. other], employment status [paid work (1) vs. other], religious background [none (1) vs. other], cultural background [Dutch (1) vs. other] and health status [very good (1) vs. other]. To answer both research questions in this study we applied mediator and moderator analyses using the PROCESS macro in SPSS (version 2.10; Hayes 2013). To assess if self-compassion as measured with the SCS-SF mediated the relationship between positive mental health as measured with the MHC-SF and psychopathology as measured with the HADS, we applied a cross-product of coefficients approach using nonparametric bootstrapping procedures (Preacher and Hayes 2004, 2008). In this approach, the cross-product of the coefficient for the relationship between MHC-SF (X) and SCS-SF (M) (the *a*-path) and the coefficient for the relationship between the SCS-SF (M) and outcome measure HADS (Y) while controlling for X (the *b*-path) was calculated based on 5000 bootstrapped samples. As can be seen from Fig. 1, this indirect effect (a*b path) is similar to the difference between the total effect (*c′*-path) and direct effect (*c*-path) of MHC (X) on HADS (Y). The overall significance of the effect of the a*b path was tested using bias-corrected 95 % confidence intervals (CI). An indirect effect was considered significant when the confidence interval did not include zero. Background dummy variables that correlated significantly with the dependent variable HADS (educational level, work and health status) controlled for in the analysis. To be able to interpret the size of the indirect effect of SCS-SF, κ^2^ was reported as a recommended standardized measure of effect size, where .01 can be interpreted as small, .09 as medium and .25 as large (Preacher and Kelley 2011).

In a second step, to assess if the SCS-SF moderated the relationship between state negative affective states as measured with the mDES and psychopathology as measured with the HADS, linear regression models were applied in PROCESS. First, scores on SCS-SF and mDES were grand mean centered to reduce possible scaling problems and multicollinearity. In the regression models, the HADS score was entered as the dependent variable followed by the centered mDES negative affect and centered SCS-SF scores as independent variables. Also included as an independent variable was an interaction effect between SCS-SF and MDES negative affect. As for the mediation analysis, background dummy variables that correlated significantly with the dependent variable HADS educational level, work and health status) were controlled for in the moderator analysis. Significance tests performed were two-tailed and corresponded with a *p* < .05. In case this interaction effect was significant, the SCS-SF was interpreted as being a moderator of the relationship between mDES and HADS. A visual representation of a potentially significant interaction effect was given, displaying the relationship between negative affect as measured with the mDES and psychopathology as measured by the HADS for three groups with different levels of self-compassion: one group with mean scores on SCS-SF, and two groups with −1 and +1 standard deviation on SCS-SF, respectively. Also simple slopes for the −1 and +1 SD groups were reported.

## Results

Intercorrelations between all measures and descriptive variables can be found in Table 2. Intercorrelations between all concepts under study (self-compassion, state negative affect, psychopathology and positive mental health) were significant and ranged from moderate to high. The lowest correlation could be observed between state negative affect and positive mental health (−), while the highest correlation was observed between state negative affect and psychopathology (−). Correlations of self-compassion with both positive mental health (+) and psychopathology (−) were significant and relatively similar. Also, positive mental health and psychopathology showed a significant, moderate correlation (−). Finally, with regard to relationship between the psychopathology outcome variable and each of the descriptive variables, significantly lower HADS scores were obtained by individuals with a high educational level, who had a paid employment status, and possessed good health. These three variables were controlled for in the remainder of the analyses (Table 3).

**Table 2:** Correlations between all measures and descriptive variables

	1.	2.	3.	4.	5.	6.	7.	8.	9.	10.	11.	12.
1. Age	–											
2. Gender	−.123^a^	–										
3. Educational level	.050	−.031	–									
4. Marital status	.679^b^	−.061	−.016	–								
5. Work activities	.331^b^	−.248^b^	.111^a^	.328^b^	–							
6. Health status	−.081	.022	.044	.019	.045	–						
7. Cultural background	.330^b^	−.149^b^	−.054	.210^b^	.179^a^	.022	–					
8. Religious background	−.093	−.050	.083	−.158^b^	−.060	.021	.319^b^	–				
9. SCS-SF	.198^b^	−.148^b^	.029	.203^b^	.213^b^	.159^b^	−.049^a^	−.066	–			
10. MHC	−.038	−.021	.089	−.046	.043	.214^b^	−.089	−.039	.493^b^	–		
11. mDES NA	−.004	.090	−.167^b^	−.010	−.117^a^	−.138^a^	−.054	−.102	−.355^b^	−.305^b^	–	
12. HADS	−.076	.064	−.145^b^	−.089	−.195^b^	−.247^b^	−.065	−.018	−.557^b^	−.448^b^	.624^b^	–

*SCS-SF* self-compassion scale—short form, *MHC* mental health continuum, *mDES NA* modified differential emotions scale—negative affect, *HADS* hospital anxiety depression scale

^a^ *p* < .05

^b^ *p* < .01

**Table 3:** Outcomes of mediation analyses using PROCESS macro (Hayes 2013)

	Coefficient	SE	*t* value	*p* value	K^2^
Total effect (c′)					
Constant	21.594	1.678	12.871	.000	
Age	−.009	.029	−.319	.750	
Marital status	−.552	.778	−.709	.480	
Educational level	−1.074	.506	−2.120	.035	
Work activities	−1.457	.537	−2.714	.007	
Health status	−1.964	.546	−3.601	.000	
MHC	−.188	.025	−7.442	.000	
Direct effect (c)					
Constant	23.673	1.578	15.002	.000	
Age	.009	.025	.378	.706	
Marital status	.010	.627	.017	.987	
Educational level	−1.207	.471	−2.562	.011	
Work activities	−.911	.486	−1.874	.062	
Health status	−1.686	.510	−3.308	.001	
SCS-SF	−.212	.293	−7.229	.000	
MHC	−.092	.025	−3.692	.000	
Indirect effect (a*b)					
SCS-SF	−.096	.017		(−.131; −.065)×	.216

Total, direct and indirect effect of the relationship between MHC and HADS with SCS-SF as potential mediator

*SE* standard error of coefficient, × significance determined by 95 % confidence intervals

First, hierarchical regression models assessing the total effect of positive mental health on psychopathology while controlling for relevant descriptive variables, showed that positive mental health was significantly and negatively related with psychopathology scores (b = −.19, t(342) = −7.44, *p* < .001). This means that in this sample higher positive mental health as measured with the MHC was related to lower psychopathology as measured with the HADS. Further regression models assessing the direct and indirect effects of self-compassion revealed a significant indirect or mediation effect (a*b) of self-compassion on psychopathology outcomes [b = −.10, 95 % CI (−.13; −.07)]. Self-compassion as measured by the SCS-SF was a significant partial mediator of the relationship between positive mental health and psychopathology. Standardized indirect effect sizes approached a large magnitude (k^2^ = .22). Overall, outcomes of mediation analyses confirmed our first hypothesis, which stated that self-compassion mediates the relationship between positive mental health and psychopathology.^1^

**Fig. 2:**
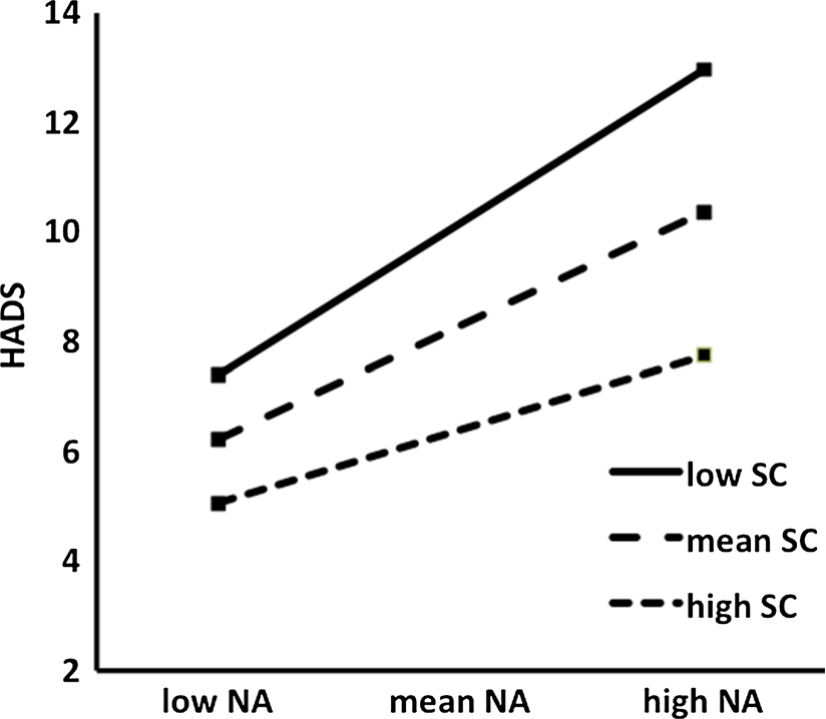
Relationship between negative affect (NA) and HADS scores at different levels of moderator SCS-SF as a measure of self-compassion (SC)

To further discern *how* self-compassion functioned as a resilience mechanism between positive mental health and psychopathology, moderation analyses were performed. We hypothesized that self-compassion would moderate the relationship between state negative affect and psychopathology, and more specifically, that for low self-compassion scores the relationship between state negative affect and psychopathology would be stronger than for higher self-compassion scores. First, the outcomes of the hierarchical regression model to test moderation showed significant direct effects of both self-compassion (b = −.18, t(340) = −8.53, *p* < .001) and state negative affect (b = .26, t(340) = 8.10, *p* < .001) on psychopathology scores. Importantly, the interaction effect between both self-compassion and state negative affect as a test of moderation was also significant (b = −.01, t(340) = −3.46, *p* < .001). In Fig. 2, the relationship between state negative affect as measured with the mDES and psychopathology as measured by the HADS are displayed for three groups with different levels of self-compassion (mean score, and ±1 SD). As can be seen from Fig. 2, for the group with +1SD self-compassion scores, the relationship between state negative affect and psychopathology is smaller (b = .16, *p* < .001) than for those with −1SD scores on self-compassion (b = .36, *p* < .001). This implies that the impact of high state negative affect as measured with the state negative affect on HADS as an indicator of psychopathology is smaller for individuals with high levels of self-compassion than for individuals with low levels of self-compassion.^2^

## Discussion

There is growing evidence that positive mental health and psychopathology are related but distinct continua, and that a higher level of positive mental health buffers against psychopathology (Grant et al. 2013; Keyes et al. 2010; Lamers et al. 2015; Wood and Joseph 2010). However, little is known about how and why this protective functioning is brought about. Such knowledge can inform theory, and guide the development of effective and efficient (preventive) interventions for positive mental health that can protect against psychopathology in the long term. We explored the role of self-compassion in explaining this protective functioning, and found preliminary evidence that *self*-*compassion* partially mediates the negative relationship between positive mental health and psychopathology. Although self-compassion did not fully mediate this relationship, the corresponding effect size of mediation approached a large effect (Preacher and Kelley 2011). This suggests that the relative contribution of self-compassion in explaining the link between positive mental health and psychopathology is substantial. Interpretation of this primary mediation model indicates that self-compassion is a resilience resource for those with high positive mental health, and explains partially why positive mental health buffers against impairment such as depressive symptoms. It must be noted, however, that we also found significant support for an alternative mediation model in which the link between *psychopathology and positive mental health* was mediated by self-compassion. This model implies that people high in depressive symptoms have an impaired ability for a self-compassionate attitude, probably due to the fact that their narrowed thinking (e.g. catastrophizing) and feeling (e.g. isolation) states make it difficult to experience the broadened and relativistic thinking and feelings states associated with self-compassion. Via this pathway depressive symptoms may impair the possibility to experience high levels of positive mental health. Overall, the fact that both mediation model were significant correspond to empirical evidence for a *bidirectional* relationship between psychopathology and positive mental health (Lamers et al. 2015), and that both depressive symptoms and/or positive mental health influence each other.

Additional outcomes suggest that self-compassion served as a moderator of the association between negative affect and psychopathology. The impact of high state negative affect on psychopathology was smaller for individuals with high levels of self-compassion than for individuals with lower levels of self-compassion. Although no causal inferences can be drawn from our data given the cross-sectional study design, our results provide support for, and build upon, findings from previous studies. We replicated a large range of studies that revealed a negative relationship between self-compassion and psychopathology in the form of depression, anxiety and stress (Barnard and Curry 2011; MacBeth and Gumley 2012), and results of a much smaller range of existing studies that suggest that self-compassion is related to aspects of positive mental health, such as positive emotions, self-acceptance, social connection, environmental mastery and purpose in life (e.g. Neff et al. 2007a; b). Our and other empirical evidence clearly indicates that self-compassion must be understood as an explicit human strength and building block of positive mental health. This study contributes to existing findings, as we operationalized positive mental health using the Mental Health Continuum (MHC) as one of the most comprehensive and theoretically most well-founded measures of positive mental health defined as optimal emotional, psychological and social well-being (Keyes 2002; Lamers et al. 2011). We additionally build upon a handful of studies that tried to investigate the specific *mechanisms* by which self-compassion might serve as a protective factor against psychopathology. Previous studies suggested that self-compassion is positively related to adaptive emotion regulation strategies, such as positive cognitive reappraisal and acceptance, and negative related to maladaptive emotion regulation strategies such as experiential avoidance, thought suppression, and rumination (e.g. Allen and Leary 2010; Barnard and Curry 2011; Leary et al. 2007). In a recent experimental study, Diedrich et al. (2014) proposed that self-compassion skills aid adaptive emotion regulation by providing a friendly, accepting and situational context for a stressor to occur in. This helps to appraise stressors as more momentary, controllable and less aversive, and subsequently prohibits the activation or generation of underlying cognitive psychopathological schema. Our findings lend further support for these ideas. Further study on the working mechanisms of self-compassion as an emotion regulation strategy is necessary.

If the findings of this study are corroborated in longitudinal studies and clinical samples it has some important implications for (positive) clinical practice. First of all, self-compassion interventions can be implemented as interventions targeting psychopathology (Hofmann et al. 2012). Compassion focused therapy and training may reduce trans-diagnostic factors, such as self-criticism or rumination, that play an important role in the development and maintenance of various psychological disorders. Several studies (e.g. Gilbert and Irons 2005; Gilbert and Procter 2006; Neff and Germer 2013) have indeed found effects of therapy and training focusing on self-compassion on psychopathology in clinical populations. Secondly, and most relevant within the theme of this special issue, enhancing self-compassion can also be seen as positive intervention enhancing positive mental health. Training self-compassion can teach important attributes such as equanimity, kindness, forgiveness, broadened awareness and feelings of inter-connectedness with others. These attributes will improve factors related to positive mental health including self-acceptance, positive social relations and positive emotions. Such positive training and intervention help people recovering from psychological disorders with future personal growth, and may provide new tools to modify the context in which future negative life-events and stressful experiences occurs. This can make people more resilient, and protects against potential future remission or the development of new mental disorders (Neff and Germer 2013). For this reason, compassion focused interventions could be important as public mental health strategies (Fledderus et al. 2010).

The cross-sectional nature of our dataset is an important limitation to this study. No causal inferences can be drawn from the cross-sectional models we tested. We have, however, given preliminary support for potential mediating and moderating mechanisms of self-compassion in relation to positive mental health, and also tested several alternative mediation and moderation models. Our proposed theoretical model as well as proposed and other alternative models need to be tested further with longitudinal designs in the future, before any strong conclusions can be articulated on the role of self-compassion as a resilience mechanism and adaptive emotion regulation strategy. Another limitation is the convenience sample used, that overrepresented younger, highly educated, and female individuals. The nature of this sample must be taken into account when considering the generalizability of the results of this study. Further studies in general public samples that are more representative of the general population need to be performed, as are studies in clinical populations. However, given that self-compassion is a relatively new concept of psychological study, it must be noted that the existing range of studies often recruited university student samples. This exploratory study shed light on self-compassion as a promising and interesting mechanism of positive mental health related to *both* positive mental health and psychopathology. Self-compassion can be understood as an important resilience mechanism for those with positive mental health that enables adaptive emotion regulation during stressful experiences in daily life and protects against the in-depth activation of psychopathological cognitive schema. In addition to a central focus on self-compassion, this study has confirmed the importance of a focus on positive mental health a significant and independent end-point of mental health intervention that functions as a highly adaptive resilience resource in adaptive human functioning. More studies on positive mechanisms of positive mental health such as self-compassion is necessary, and may help to enhance our therapeutic interventions for both clinical populations and general public health in the future.

## Footnotes

1 We also ran an alternative mediation model. We found significant, partial mediation for the link between psychopathology and positive mental health as mediated by self-compassion [indirect effect = −.447, 95 % CI = (−.607; −.312)]. The findings correspond to empirical evidence for a *bidirectional* relationship between psychopathology and positive mental health (Lamers et al. 2015).

2 We also ran an alternative moderation model. We found no evidence for a model in which self-compassion would moderate the relationship between psychopathology and state negative affect (interaction effect = −.006, SE = .005, *p* = .193).
